# (Acetato-κ*O*){bis­[(2,4-dimethyl-1*H*-pyrazol-1-yl)meth­yl][(pyridin-2-yl)meth­yl]amine}­cobalt(II) hexa­fluorido­phosphate

**DOI:** 10.1107/S1600536812035222

**Published:** 2012-09-08

**Authors:** Fan Yu

**Affiliations:** a Key Laboratory of Optoelectronic Chemical Materials and Devices, of the Ministry of Education, Jianghan University, Wuhan 430056, People’s Republic of China; b School of Chemical and Environmental Engineering, Jianghan University, Wuhan 430056, People’s Republic of China

## Abstract

In the title compound, [Co(CH_3_CO_2_)(C_18_H_24_N_6_)]PF_6_, the Co^II^ atom is penta­coordinated in a distorted trigonal–bipyramidal geometry by four N atoms from a tripodal ligand and one O atom from a monodentate acetate ligand. The crystal packing is stabilized by inter­molecular C—H⋯F and C—H⋯O hydrogen bonds.

## Related literature

For related structures, see: Kumar *et al.* (2012 ▶); Li *et al.* (2008 ▶); Tao *et al.* (2006 ▶).
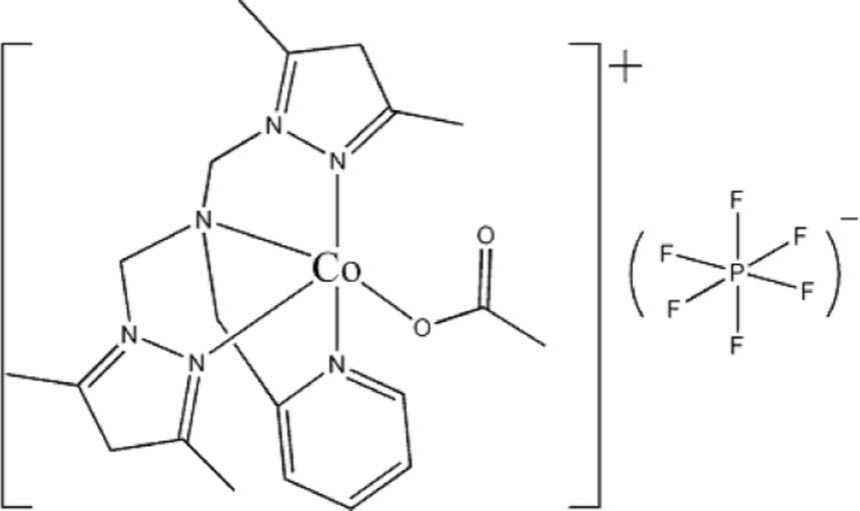


## Experimental

### 

#### Crystal data

[Co(C_2_H_3_O_2_)(C_18_H_24_N_6_)]PF_6_
*M* *_r_* = 587.38
Monoclinic, 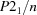

*a* = 13.7489 (6) Å
*b* = 13.0185 (5) Å
*c* = 15.4765 (7) Å
β = 115.759 (6)°
*V* = 2494.9 (2) Å^3^
*Z* = 4
Mo *K*α radiation
μ = 0.83 mm^−1^
*T* = 293 K
0.25 × 0.20 × 0.20 mm

#### Data collection

Oxford Diffraction Gemini S Ultra diffractometer
Absorption correction: multi-scan (*CrysAlis RED*; Oxford Diffraction, 2006 ▶) *T*_min_ = 0.820, *T*_max_ = 0.852
19458 measured reflections
4890 independent reflections
3136 reflections with *I* > 2σ(*I*)
*R*_int_ = 0.056

#### Refinement

*R*[*F* ^2^ > 2σ(*F* ^2^)] = 0.051
*wR*(*F* ^2^) = 0.129
*S* = 0.97
4890 reflections
325 parameters
H-atom parameters constrained
Δρ_max_ = 0.76 e Å^−3^
Δρ_min_ = −0.39 e Å^−3^

### 

Data collection: *CrysAlis CCD* (Oxford Diffraction, 2006 ▶); cell refinement: *CrysAlis RED* (Oxford Diffraction, 2006 ▶); data reduction: *CrysAlis RED*; program(s) used to solve structure: *SHELXS97* (Sheldrick, 2008 ▶); program(s) used to refine structure: *SHELXL97* (Sheldrick, 2008 ▶); molecular graphics: *XP* in *SHELXTL* (Sheldrick, 2008 ▶); software used to prepare material for publication: *SHELXTL*.

## Supplementary Material

Crystal structure: contains datablock(s) I, global. DOI: 10.1107/S1600536812035222/hy2569sup1.cif

Structure factors: contains datablock(s) I. DOI: 10.1107/S1600536812035222/hy2569Isup2.hkl

Additional supplementary materials:  crystallographic information; 3D view; checkCIF report

## Figures and Tables

**Table 1:** Hydrogen-bond geometry (Å, °)

*D*—H⋯*A*	*D*—H	H⋯*A*	*D*⋯*A*	*D*—H⋯*A*
C14—H14*A*⋯F2	0.97	2.52	3.309 (5)	139
C14—H14*A*⋯F3	0.97	2.45	3.234 (5)	138
C22—H22*A*⋯F6^i^	0.93	2.47	3.403 (6)	175
C24—H24*C*⋯O2^ii^	0.96	2.59	3.501 (5)	159
C25—H25*A*⋯F4^iii^	0.97	2.44	3.288 (5)	146
C32—H32*C*⋯O2^iv^	0.96	2.57	3.300 (5)	133

Symmetry codes: (i) 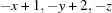; (ii) 
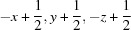
; (iii) 
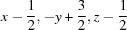
; (iv) 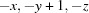.

**Fig. 1.**
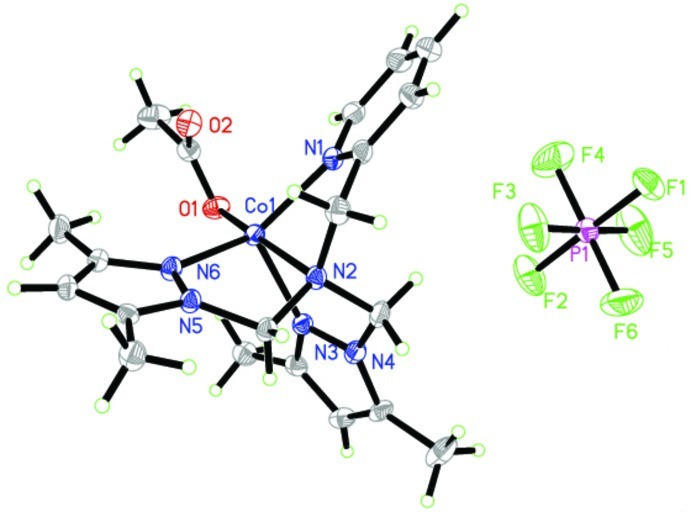
The molecular structure of the title compound. Displacement ellipsoids are drawn at the 30% probability level.

## supplementary crystallographic information

### Comment

Mononuclear metal complexes containing heterocyclic ligands, such as pyrazole and imidazole, have been the subject of active areas of research because these molecules mimic the active coordination sites of metalloproteins (Kumar *et al.*, 2012) or act as the precursor of functional complexes (Li *et al.*, 2008). To study new transition metal complexes with biologically active pyrazole-based ligands, we have synthesized the title compound.

In the title compound, the Co^II^ atom is pentacoordinated in a distorted trigonal-bipyramidal geometry by four N atoms from a tripodal ligand and one O atom from a monodentate acetate ligand (Fig. 1), with an average Co—N distance of 2.154 (2) Å and a Co—O distance of 2.003 (2) Å. The bond lengths and angles are in consistent with the typical values for the Co(II) complexes (Tao *et al.*, 2006). The crystal packing is stabilized by intermolecular C—H···F and C—H···O hydrogen bonds (Table 1).

### Experimental

To a well stirred methanol solution (20 ml) containing the corresponding tripodal ligand (2.02 mmol) and Co(CH_3_COO)_2_.6H_2_O (2.0 mmol) was added an aqueous solution (10 ml) of KPF_6_ (5.0 mmol). Green crystals of the title compound were obtained from the resulting filtrate. The products were filtered by sanction, washed with water and methanol for several times.

### Refinement

H atoms were positioned geometrically and refined as riding atoms, with C—H = 0.93 (aromatic), 0.97 (CH_2_) and 0.96 (CH_3_) Å and with *U*_iso_(H) = 1.2(1.5 for methyl)*U*_eq_(C).

### Crystal data

**Table:**

[Co(C_2_H_3_O_2_)(C_18_H_24_N_6_)]PF_6_	*F*(000) = 1204
*M**_r_* = 587.38	*D*_x_ = 1.564 Mg m^−^^3^*D*_m_ = 1.564 Mg m^−^^3^*D*_m_ measured by not measured
Monoclinic, *P*2_1_/*n*	Mo *K*α radiation, λ = 0.71073 Å
Hall symbol: -P 2yn	Cell parameters from 4897 reflections
*a* = 13.7489 (6) Å	θ = 2.1–26.0°
*b* = 13.0185 (5) Å	µ = 0.83 mm^−^^1^
*c* = 15.4765 (7) Å	*T* = 293 K
β = 115.759 (6)°	Block, green
*V* = 2494.9 (2) Å^3^	0.25 × 0.20 × 0.20 mm
*Z* = 4	

### Data collection

**Table:**

Oxford Diffraction Gemini S Ultra diffractometer	4890 independent reflections
Radiation source: fine-focus sealed tube	3136 reflections with *I* > 2σ(*I*)
Graphite monochromator	*R*_int_ = 0.056
Detector resolution: 0 pixels mm^-1^	θ_max_ = 26.0°, θ_min_ = 2.1°
ω scans	*h* = −16→16
Absorption correction: multi-scan (*CrysAlis RED*; Oxford Diffraction, 2006)	*k* = −16→15
*T*_min_ = 0.820, *T*_max_ = 0.852	*l* = −18→19
19458 measured reflections	

### Refinement

**Table:**

Refinement on *F*^2^	Primary atom site location: structure-invariant direct methods
Least-squares matrix: full	Secondary atom site location: difference Fourier map
*R*[*F*^2^ > 2σ(*F*^2^)] = 0.051	Hydrogen site location: inferred from neighbouring sites
*wR*(*F*^2^) = 0.129	H-atom parameters constrained
*S* = 0.97	*w* = 1/[σ^2^(*F*_o_^2^) + (0.069*P*)^2^] where *P* = (*F*_o_^2^ + 2*F*_c_^2^)/3
4890 reflections	(Δ/σ)_max_ = 0.001
325 parameters	Δρ_max_ = 0.76 e Å^−^^3^
0 restraints	Δρ_min_ = −0.39 e Å^−^^3^

### Special details

**Table:**

Geometry. All e.s.d.'s (except the e.s.d. in the dihedral angle between two l.s. planes) are estimated using the full covariance matrix. The cell e.s.d.'s are taken into account individually in the estimation of e.s.d.'s in distances, angles and torsion angles; correlations between e.s.d.'s in cell parameters are only used when they are defined by crystal symmetry. An approximate (isotropic) treatment of cell e.s.d.'s is used for estimating e.s.d.'s involving l.s. planes.
Refinement. Refinement of *F*^2^ against ALL reflections. The weighted *R*-factor *wR* and goodness of fit *S* are based on *F*^2^, conventional *R*-factors *R* are based on *F*, with *F* set to zero for negative *F*^2^. The threshold expression of *F*^2^ > σ(*F*^2^) is used only for calculating *R*-factors(gt) *etc*. and is not relevant to the choice of reflections for refinement. *R*-factors based on *F*^2^ are statistically about twice as large as those based on *F*, and *R*- factors based on ALL data will be even larger.

### Fractional atomic coordinates and isotropic or equivalent isotropic displacement parameters (Å^2^)

**Table:**

	*x*	*y*	*z*	*U*_iso_*/*U*_eq_	
Co1	0.21796 (4)	0.72452 (4)	0.09330 (3)	0.02612 (16)	
O1	0.1832 (2)	0.7956 (2)	0.19132 (18)	0.0382 (7)	
O2	0.1668 (2)	0.6300 (2)	0.20698 (19)	0.0392 (7)	
N2	0.2747 (2)	0.6622 (2)	−0.0162 (2)	0.0273 (7)	
N3	0.2597 (2)	0.8535 (2)	0.0399 (2)	0.0287 (7)	
N1	0.3582 (2)	0.6391 (2)	0.1721 (2)	0.0311 (8)	
N4	0.3193 (2)	0.8376 (3)	−0.0096 (2)	0.0320 (8)	
C8	0.1624 (3)	0.7190 (4)	0.2326 (3)	0.0350 (9)	
N6	0.0781 (2)	0.6710 (2)	−0.0169 (2)	0.0290 (7)	
N5	0.0880 (2)	0.6312 (2)	−0.0950 (2)	0.0276 (7)	
C11	−0.0037 (3)	0.5858 (3)	−0.1566 (2)	0.0286 (9)	
C12	−0.0753 (3)	0.5944 (3)	−0.1181 (3)	0.0358 (10)	
H12A	−0.1457	0.5697	−0.1443	0.043*	
C14	0.3562 (3)	0.7344 (3)	−0.0146 (3)	0.0367 (10)	
H14A	0.4243	0.7212	0.0406	0.044*	
H14B	0.3671	0.7266	−0.0721	0.044*	
C16	0.3396 (3)	0.9270 (3)	−0.0439 (3)	0.0385 (11)	
C17	−0.0226 (3)	0.6476 (3)	−0.0316 (3)	0.0309 (9)	
C18	0.5017 (4)	0.5877 (4)	0.3187 (3)	0.0523 (12)	
H18A	0.5413	0.5969	0.3845	0.063*	
C19	0.3871 (3)	0.5661 (3)	0.1266 (3)	0.0304 (9)	
C20	0.3210 (3)	0.5617 (3)	0.0205 (3)	0.0380 (10)	
H20A	0.3659	0.5399	−0.0100	0.046*	
H20B	0.2635	0.5117	0.0054	0.046*	
C22	0.2900 (3)	1.0021 (3)	−0.0151 (3)	0.0375 (10)	
H22A	0.2892	1.0721	−0.0275	0.045*	
C23	0.4712 (3)	0.5003 (3)	0.1747 (3)	0.0386 (10)	
H23A	0.4893	0.4490	0.1424	0.046*	
C24	0.1742 (3)	1.0008 (3)	0.0794 (3)	0.0415 (10)	
H24A	0.1512	0.9482	0.1100	0.062*	
H24B	0.1120	1.0331	0.0302	0.062*	
H24C	0.2160	1.0511	0.1262	0.062*	
C25	0.1813 (3)	0.6592 (3)	−0.1093 (2)	0.0304 (9)	
H25A	0.1704	0.7259	−0.1400	0.036*	
H25B	0.1929	0.6092	−0.1504	0.036*	
C27	0.4164 (3)	0.6493 (3)	0.2672 (3)	0.0422 (11)	
H27A	0.3976	0.7006	0.2990	0.051*	
C29	−0.0650 (3)	0.6777 (4)	0.0380 (3)	0.0454 (11)	
H29A	−0.0098	0.7133	0.0909	0.068*	
H29B	−0.0864	0.6174	0.0610	0.068*	
H29C	−0.1263	0.7220	0.0070	0.068*	
C30	0.2412 (3)	0.9542 (3)	0.0360 (2)	0.0326 (9)	
C31	0.1285 (4)	0.7417 (4)	0.3103 (3)	0.0557 (13)	
H31A	0.1158	0.6784	0.3356	0.083*	
H31B	0.0633	0.7817	0.2845	0.083*	
H31C	0.1845	0.7796	0.3606	0.083*	
C32	−0.0137 (3)	0.5399 (3)	−0.2481 (3)	0.0400 (10)	
H32A	0.0538	0.5466	−0.2522	0.060*	
H32B	−0.0692	0.5749	−0.3012	0.060*	
H32C	−0.0320	0.4685	−0.2501	0.060*	
C33	0.5278 (4)	0.5124 (4)	0.2721 (3)	0.0517 (12)	
H33A	0.5849	0.4684	0.3066	0.062*	
C34	0.4055 (4)	0.9310 (4)	−0.0981 (3)	0.0571 (14)	
H34A	0.4268	0.8626	−0.1058	0.086*	
H34B	0.4687	0.9720	−0.0637	0.086*	
H34C	0.3638	0.9608	−0.1601	0.086*	
P1	0.69225 (8)	0.70775 (8)	0.13886 (8)	0.0368 (3)	
F1	0.7752 (2)	0.6167 (2)	0.15406 (17)	0.0589 (7)	
F2	0.6097 (2)	0.7997 (2)	0.1226 (2)	0.0752 (9)	
F3	0.5946 (2)	0.6330 (2)	0.0763 (2)	0.0796 (10)	
F4	0.6815 (3)	0.6735 (3)	0.2316 (2)	0.0923 (11)	
F5	0.7866 (3)	0.7796 (3)	0.2033 (3)	0.1060 (13)	
F6	0.7016 (3)	0.7399 (3)	0.0464 (3)	0.1068 (13)	

### Atomic displacement parameters (Å^2^)

**Table:**

	*U*^11^	*U*^22^	*U*^33^	*U*^12^	*U*^13^	*U*^23^
Co1	0.0261 (3)	0.0290 (3)	0.0229 (3)	−0.0040 (2)	0.0103 (2)	−0.0024 (2)
O1	0.0510 (17)	0.0346 (17)	0.0341 (15)	0.0009 (14)	0.0231 (14)	0.0000 (12)
O2	0.0412 (16)	0.0356 (18)	0.0438 (16)	−0.0021 (13)	0.0211 (14)	−0.0069 (13)
N2	0.0231 (16)	0.0316 (19)	0.0258 (16)	−0.0044 (14)	0.0093 (13)	−0.0033 (13)
N3	0.0262 (17)	0.033 (2)	0.0258 (16)	−0.0050 (14)	0.0100 (14)	−0.0013 (14)
N1	0.0248 (16)	0.035 (2)	0.0309 (18)	−0.0048 (15)	0.0095 (14)	0.0015 (15)
N4	0.0297 (17)	0.039 (2)	0.0273 (17)	−0.0063 (16)	0.0128 (15)	−0.0020 (15)
C8	0.035 (2)	0.043 (3)	0.028 (2)	0.004 (2)	0.0141 (18)	−0.001 (2)
N6	0.0283 (17)	0.0369 (19)	0.0254 (16)	−0.0029 (15)	0.0149 (14)	−0.0038 (14)
N5	0.0250 (16)	0.0351 (19)	0.0242 (16)	−0.0030 (14)	0.0121 (14)	−0.0025 (14)
C11	0.027 (2)	0.033 (2)	0.0201 (18)	−0.0039 (18)	0.0045 (16)	0.0027 (16)
C12	0.025 (2)	0.046 (3)	0.033 (2)	−0.0070 (19)	0.0093 (17)	0.0034 (19)
C14	0.029 (2)	0.049 (3)	0.037 (2)	−0.008 (2)	0.0192 (18)	−0.002 (2)
C16	0.037 (2)	0.050 (3)	0.023 (2)	−0.026 (2)	0.0076 (18)	0.0008 (19)
C17	0.027 (2)	0.034 (2)	0.031 (2)	−0.0021 (18)	0.0127 (18)	0.0024 (18)
C18	0.040 (3)	0.066 (3)	0.043 (3)	−0.007 (3)	0.010 (2)	0.016 (2)
C19	0.025 (2)	0.029 (2)	0.037 (2)	−0.0042 (17)	0.0132 (17)	−0.0003 (18)
C20	0.034 (2)	0.041 (3)	0.035 (2)	−0.005 (2)	0.0122 (19)	−0.0072 (19)
C22	0.044 (2)	0.032 (2)	0.029 (2)	−0.016 (2)	0.008 (2)	−0.0007 (18)
C23	0.033 (2)	0.034 (2)	0.047 (3)	0.0022 (19)	0.016 (2)	0.004 (2)
C24	0.044 (2)	0.036 (3)	0.042 (2)	0.000 (2)	0.016 (2)	−0.0003 (19)
C25	0.026 (2)	0.042 (2)	0.0239 (19)	−0.0047 (18)	0.0116 (17)	−0.0029 (17)
C27	0.035 (2)	0.052 (3)	0.035 (2)	−0.009 (2)	0.012 (2)	0.010 (2)
C29	0.032 (2)	0.067 (3)	0.040 (2)	−0.002 (2)	0.018 (2)	0.000 (2)
C30	0.031 (2)	0.032 (2)	0.024 (2)	−0.0073 (18)	0.0017 (17)	−0.0019 (17)
C31	0.085 (4)	0.053 (3)	0.046 (3)	0.015 (3)	0.043 (3)	0.004 (2)
C32	0.036 (2)	0.052 (3)	0.030 (2)	−0.009 (2)	0.0133 (18)	−0.0087 (19)
C33	0.039 (3)	0.051 (3)	0.062 (3)	0.006 (2)	0.020 (2)	0.022 (3)
C34	0.067 (3)	0.070 (4)	0.043 (3)	−0.036 (3)	0.032 (2)	−0.012 (2)
P1	0.0359 (6)	0.0387 (7)	0.0379 (6)	0.0048 (5)	0.0180 (5)	0.0024 (5)
F1	0.0548 (16)	0.0618 (18)	0.0536 (16)	0.0247 (14)	0.0175 (13)	0.0043 (13)
F2	0.0545 (17)	0.0558 (19)	0.101 (2)	0.0191 (14)	0.0206 (17)	−0.0136 (16)
F3	0.0486 (17)	0.066 (2)	0.113 (2)	−0.0015 (15)	0.0257 (17)	−0.0362 (18)
F4	0.140 (3)	0.093 (2)	0.084 (2)	0.008 (2)	0.085 (2)	0.0041 (19)
F5	0.0547 (19)	0.074 (2)	0.152 (3)	−0.0146 (18)	0.010 (2)	−0.037 (2)
F6	0.134 (3)	0.131 (3)	0.087 (2)	0.051 (3)	0.078 (2)	0.060 (2)

### Geometric parameters (Å, º)

**Table:**

Co1—O1	2.004 (3)	C19—C20	1.493 (5)
Co1—N6	2.059 (3)	C20—H20A	0.9700
Co1—N3	2.060 (3)	C20—H20B	0.9700
Co1—N1	2.097 (3)	C22—C30	1.388 (5)
Co1—N2	2.298 (3)	C22—H22A	0.9300
O1—C8	1.281 (5)	C23—C33	1.373 (6)
O2—C8	1.234 (5)	C23—H23A	0.9300
N2—C14	1.455 (5)	C24—C30	1.485 (5)
N2—C25	1.456 (4)	C24—H24A	0.9600
N2—C20	1.458 (5)	C24—H24B	0.9600
N3—C30	1.332 (5)	C24—H24C	0.9600
N3—N4	1.359 (4)	C25—H25A	0.9700
N1—C19	1.342 (5)	C25—H25B	0.9700
N1—C27	1.342 (5)	C27—H27A	0.9300
N4—C16	1.357 (5)	C29—H29A	0.9600
N4—C14	1.450 (5)	C29—H29B	0.9600
C8—C31	1.497 (5)	C29—H29C	0.9600
N6—C17	1.337 (5)	C31—H31A	0.9600
N6—N5	1.375 (4)	C31—H31B	0.9600
N5—C11	1.344 (4)	C31—H31C	0.9600
N5—C25	1.441 (4)	C32—H32A	0.9600
C11—C12	1.358 (5)	C32—H32B	0.9600
C11—C32	1.488 (5)	C32—H32C	0.9600
C12—C17	1.398 (5)	C33—H33A	0.9300
C12—H12A	0.9300	C34—H34A	0.9600
C14—H14A	0.9700	C34—H34B	0.9600
C14—H14B	0.9700	C34—H34C	0.9600
C16—C22	1.373 (6)	P1—F6	1.549 (3)
C16—C34	1.480 (6)	P1—F5	1.558 (3)
C17—C29	1.483 (5)	P1—F4	1.570 (3)
C18—C33	1.355 (7)	P1—F1	1.590 (3)
C18—C27	1.357 (6)	P1—F2	1.592 (3)
C18—H18A	0.9300	P1—F3	1.600 (3)
C19—C23	1.369 (5)		
			
O1—Co1—N6	109.80 (11)	C16—C22—H22A	126.4
O1—Co1—N3	97.31 (12)	C30—C22—H22A	126.4
N6—Co1—N3	105.45 (12)	C19—C23—C33	117.8 (4)
O1—Co1—N1	105.43 (12)	C19—C23—H23A	121.1
N6—Co1—N1	126.43 (12)	C33—C23—H23A	121.1
N3—Co1—N1	108.63 (12)	C30—C24—H24A	109.5
O1—Co1—N2	171.77 (11)	C30—C24—H24B	109.5
N6—Co1—N2	76.38 (11)	H24A—C24—H24B	109.5
N3—Co1—N2	75.47 (12)	C30—C24—H24C	109.5
N1—Co1—N2	73.78 (11)	H24A—C24—H24C	109.5
C8—O1—Co1	101.3 (2)	H24B—C24—H24C	109.5
C14—N2—C25	112.0 (3)	N5—C25—N2	108.3 (3)
C14—N2—C20	111.7 (3)	N5—C25—H25A	110.0
C25—N2—C20	114.1 (3)	N2—C25—H25A	110.0
C14—N2—Co1	105.4 (2)	N5—C25—H25B	110.0
C25—N2—Co1	107.7 (2)	N2—C25—H25B	110.0
C20—N2—Co1	105.1 (2)	H25A—C25—H25B	108.4
C30—N3—N4	105.9 (3)	N1—C27—C18	122.5 (4)
C30—N3—Co1	137.6 (3)	N1—C27—H27A	118.7
N4—N3—Co1	116.3 (2)	C18—C27—H27A	118.7
C19—N1—C27	118.2 (3)	C17—C29—H29A	109.5
C19—N1—Co1	118.6 (2)	C17—C29—H29B	109.5
C27—N1—Co1	123.0 (3)	H29A—C29—H29B	109.5
C16—N4—N3	111.5 (3)	C17—C29—H29C	109.5
C16—N4—C14	129.6 (3)	H29A—C29—H29C	109.5
N3—N4—C14	118.8 (3)	H29B—C29—H29C	109.5
O2—C8—O1	121.2 (4)	N3—C30—C22	109.7 (4)
O2—C8—C31	121.3 (4)	N3—C30—C24	121.7 (3)
O1—C8—C31	117.5 (4)	C22—C30—C24	128.5 (4)
C17—N6—N5	104.8 (3)	C8—C31—H31A	109.5
C17—N6—Co1	138.1 (2)	C8—C31—H31B	109.5
N5—N6—Co1	116.2 (2)	H31A—C31—H31B	109.5
C11—N5—N6	111.7 (3)	C8—C31—H31C	109.5
C11—N5—C25	128.7 (3)	H31A—C31—H31C	109.5
N6—N5—C25	118.4 (3)	H31B—C31—H31C	109.5
N5—C11—C12	106.7 (3)	C11—C32—H32A	109.5
N5—C11—C32	121.5 (3)	C11—C32—H32B	109.5
C12—C11—C32	131.8 (3)	H32A—C32—H32B	109.5
C11—C12—C17	106.8 (3)	C11—C32—H32C	109.5
C11—C12—H12A	126.6	H32A—C32—H32C	109.5
C17—C12—H12A	126.6	H32B—C32—H32C	109.5
N4—C14—N2	108.3 (3)	C18—C33—C23	120.9 (4)
N4—C14—H14A	110.0	C18—C33—H33A	119.6
N2—C14—H14A	110.0	C23—C33—H33A	119.6
N4—C14—H14B	110.0	C16—C34—H34A	109.5
N2—C14—H14B	110.0	C16—C34—H34B	109.5
H14A—C14—H14B	108.4	H34A—C34—H34B	109.5
N4—C16—C22	105.6 (3)	C16—C34—H34C	109.5
N4—C16—C34	122.1 (4)	H34A—C34—H34C	109.5
C22—C16—C34	132.3 (4)	H34B—C34—H34C	109.5
N6—C17—C12	110.1 (3)	F6—P1—F5	92.8 (2)
N6—C17—C29	121.4 (3)	F6—P1—F4	179.0 (2)
C12—C17—C29	128.5 (3)	F5—P1—F4	88.2 (2)
C33—C18—C27	118.4 (4)	F6—P1—F1	89.78 (17)
C33—C18—H18A	120.8	F5—P1—F1	90.48 (17)
C27—C18—H18A	120.8	F4—P1—F1	89.95 (17)
N1—C19—C23	122.1 (4)	F6—P1—F2	89.42 (18)
N1—C19—C20	115.2 (3)	F5—P1—F2	89.31 (17)
C23—C19—C20	122.7 (4)	F4—P1—F2	90.85 (18)
N2—C20—C19	110.4 (3)	F1—P1—F2	179.16 (18)
N2—C20—H20A	109.6	F6—P1—F3	89.5 (2)
C19—C20—H20A	109.6	F5—P1—F3	177.7 (2)
N2—C20—H20B	109.6	F4—P1—F3	89.55 (19)
C19—C20—H20B	109.6	F1—P1—F3	89.76 (15)
H20A—C20—H20B	108.1	F2—P1—F3	90.48 (15)
C16—C22—C30	107.2 (4)		
			
N6—Co1—O1—C8	74.4 (2)	N6—N5—C11—C32	178.1 (3)
N3—Co1—O1—C8	−176.2 (2)	C25—N5—C11—C32	11.1 (6)
N1—Co1—O1—C8	−64.5 (2)	N5—C11—C12—C17	0.5 (4)
N6—Co1—N2—C14	141.2 (2)	C32—C11—C12—C17	−178.2 (4)
N3—Co1—N2—C14	31.0 (2)	C16—N4—C14—N2	−150.2 (3)
N1—Co1—N2—C14	−83.8 (2)	N3—N4—C14—N2	33.6 (4)
N6—Co1—N2—C25	21.5 (2)	C25—N2—C14—N4	75.6 (4)
N3—Co1—N2—C25	−88.8 (2)	C20—N2—C14—N4	−154.8 (3)
N1—Co1—N2—C25	156.4 (3)	Co1—N2—C14—N4	−41.2 (3)
N6—Co1—N2—C20	−100.6 (2)	N3—N4—C16—C22	−0.4 (4)
N3—Co1—N2—C20	149.2 (2)	C14—N4—C16—C22	−176.8 (3)
N1—Co1—N2—C20	34.4 (2)	N3—N4—C16—C34	178.4 (3)
O1—Co1—N3—C30	−22.3 (4)	C14—N4—C16—C34	1.9 (6)
N6—Co1—N3—C30	90.7 (4)	N5—N6—C17—C12	−0.4 (4)
N1—Co1—N3—C30	−131.3 (4)	Co1—N6—C17—C12	−167.9 (3)
N2—Co1—N3—C30	161.7 (4)	N5—N6—C17—C29	180.0 (3)
O1—Co1—N3—N4	161.1 (2)	Co1—N6—C17—C29	12.5 (6)
N6—Co1—N3—N4	−85.9 (2)	C11—C12—C17—N6	−0.1 (5)
N1—Co1—N3—N4	52.1 (2)	C11—C12—C17—C29	179.5 (4)
N2—Co1—N3—N4	−14.9 (2)	C27—N1—C19—C23	2.2 (5)
O1—Co1—N1—C19	165.4 (3)	Co1—N1—C19—C23	−173.4 (3)
N6—Co1—N1—C19	35.6 (3)	C27—N1—C19—C20	−178.0 (3)
N3—Co1—N1—C19	−91.2 (3)	Co1—N1—C19—C20	6.4 (4)
N2—Co1—N1—C19	−23.1 (3)	C14—N2—C20—C19	72.0 (4)
O1—Co1—N1—C27	−10.0 (3)	C25—N2—C20—C19	−159.6 (3)
N6—Co1—N1—C27	−139.8 (3)	Co1—N2—C20—C19	−41.8 (3)
N3—Co1—N1—C27	93.4 (3)	N1—C19—C20—N2	26.5 (5)
N2—Co1—N1—C27	161.5 (3)	C23—C19—C20—N2	−153.7 (3)
C30—N3—N4—C16	0.6 (4)	N4—C16—C22—C30	0.0 (4)
Co1—N3—N4—C16	178.2 (2)	C34—C16—C22—C30	−178.5 (4)
C30—N3—N4—C14	177.5 (3)	N1—C19—C23—C33	−1.4 (6)
Co1—N3—N4—C14	−4.9 (4)	C20—C19—C23—C33	178.8 (4)
Co1—O1—C8—O2	−0.3 (4)	C11—N5—C25—N2	−154.8 (3)
Co1—O1—C8—C31	−178.0 (3)	N6—N5—C25—N2	39.0 (4)
O1—Co1—N6—C17	−20.9 (4)	C14—N2—C25—N5	−151.6 (3)
N3—Co1—N6—C17	−124.8 (4)	C20—N2—C25—N5	80.1 (4)
N1—Co1—N6—C17	107.2 (4)	Co1—N2—C25—N5	−36.1 (3)
N2—Co1—N6—C17	164.7 (4)	C19—N1—C27—C18	−1.1 (6)
O1—Co1—N6—N5	172.6 (2)	Co1—N1—C27—C18	174.3 (3)
N3—Co1—N6—N5	68.7 (3)	C33—C18—C27—N1	−0.8 (6)
N1—Co1—N6—N5	−59.3 (3)	N4—N3—C30—C22	−0.6 (4)
N2—Co1—N6—N5	−1.8 (2)	Co1—N3—C30—C22	−177.4 (3)
C17—N6—N5—C11	0.7 (4)	N4—N3—C30—C24	177.6 (3)
Co1—N6—N5—C11	171.5 (2)	Co1—N3—C30—C24	0.8 (6)
C17—N6—N5—C25	169.2 (3)	C16—C22—C30—N3	0.4 (4)
Co1—N6—N5—C25	−20.0 (4)	C16—C22—C30—C24	−177.7 (4)
N6—N5—C11—C12	−0.8 (4)	C27—C18—C33—C23	1.6 (7)
C25—N5—C11—C12	−167.8 (4)	C19—C23—C33—C18	−0.6 (6)

### Hydrogen-bond geometry (Å, º)

**Table:**

*D*—H···*A*	*D*—H	H···*A*	*D*···*A*	*D*—H···*A*
C14—H14*A*···F2	0.97	2.52	3.309 (5)	139
C14—H14*A*···F3	0.97	2.45	3.234 (5)	138
C22—H22*A*···F6^i^	0.93	2.47	3.403 (6)	175
C24—H24*C*···O2^ii^	0.96	2.59	3.501 (5)	159
C25—H25*A*···F4^iii^	0.97	2.44	3.288 (5)	146
C32—H32*C*···O2^iv^	0.96	2.57	3.300 (5)	133

Symmetry codes: (i) −*x*+1, −*y*+2, −*z*; (ii) −*x*+1/2, *y*+1/2, −*z*+1/2; (iii) *x*−1/2, −*y*+3/2, *z*−1/2; (iv) −*x*, −*y*+1, −*z*.
